# Questionnaire-based study to assess the association between management practices and mastitis within tie-stall and free-stall dairy housing systems in Switzerland

**DOI:** 10.1186/1746-6148-9-200

**Published:** 2013-10-09

**Authors:** Paz F Gordon, Bart HP van den Borne, Martin Reist, Samuel Kohler, Marcus G Doherr

**Affiliations:** 1 Veterinary Public Health Institute, Vetsuisse Faculty, University of Bern, Schwarzenburgstrasse 155, CH-3097 Bern, Switzerland; 2 School of Agricultural, Forest and Food Sciences, Bern University of Applied Science, Länggasse 85, CH-3052 Zollikofen, Switzerland

**Keywords:** Cumulative incidence, Prevalence, Swiss dairy farms, Clinical mastitis, Subclinical mastitis, Prophylaxis, Management, Negative binomial regression, Somatic cell count

## Abstract

**Background:**

Prophylactic measures are key components of dairy herd mastitis control programs, but some are only relevant in specific housing systems. To assess the association between management practices and mastitis incidence, data collected in 2011 by a survey among 979 randomly selected Swiss dairy farms, and information from the regular test day recordings from 680 of these farms was analyzed.

**Results:**

The median incidence of farmer-reported clinical mastitis (ICM) was 11.6 (mean 14.7) cases per 100 cows per year. The median annual proportion of milk samples with a composite somatic cell count (PSCC) above 200,000 cells/ml was 16.1 (mean 17.3) %. A multivariable negative binomial regression model was fitted for each of the mastitis indicators for farms with tie-stall and free-stall housing systems separately to study the effect of other (than housing system) management practices on the ICM and PSCC events (above 200,000 cells/ml). The results differed substantially by housing system and outcome. In tie-stall systems, clinical mastitis incidence was mainly affected by region (mountainous production zone; incidence rate ratio (IRR) = 0.73), the dairy herd replacement system (1.27) and farmers age (0.81). The proportion of high SCC was mainly associated with dry cow udder controls (IRR = 0.67), clean bedding material at calving (IRR = 1.72), using total merit values to select bulls (IRR = 1.57) and body condition scoring (IRR = 0.74). In free-stall systems, the IRR for clinical mastitis was mainly associated with stall climate/temperature (IRR = 1.65), comfort mats as resting surface (IRR = 0.75) and when no feed analysis was carried out (IRR = 1.18). The proportion of high SSC was only associated with hand and arm cleaning after calving (IRR = 0.81) and beef producing value to select bulls (IRR = 0.66).

**Conclusions:**

There were substantial differences in identified risk factors in the four models. Some of the factors were in agreement with the reported literature while others were not. This highlights the multifactorial nature of the disease and the differences in the risks for both mastitis manifestations. Attempting to understand these multifactorial associations for mastitis within larger management groups continues to play an important role in mastitis control programs.

## Background

Animal health in dairy herds has clearly improved in the last decades. Disease prophylactic measures and improved management practices have played a key role in this success [1]. Nevertheless, production diseases such as mastitis still are of major concern and account for substantial economic losses for dairy farmers [2-4].

Various incidence estimates of clinical mastitis (ICM) in dairy cows have been reported by others before [5]. They are known to be influenced by factors such as genetics, nutrition and management practices [6]. In the past three decades, various studies have looked for associations between management practices on dairy farms and herd-level somatic cell counts (SCC) [7], but the majority were conducted without taking into account the possible influence of the different type of housing systems on the incidence of mastitis.

A comparison between disease occurrence in Norwegian dairy herds with a free-stall or a tie-stall barn revealed a significantly higher ICM in tie-stall barns [8]. In addition, a study carried out in Sweden which analysed the effects of changes in housing system showed that the occurrence of clinical mastitis and teat injuries decreased when changing from tie-stalls to cubicle or straw-yard housing systems [9]. A further study carried out in Canada [5] also revealed that *Staphylococcus aureus* and streptococci ICM were higher in tie-stall barns while *Escherichia coli* ICM was higher in free-stall barns.

Tie-stall systems are still the most abundant type of housing system in Switzerland, and farmers with these systems tend to use bucket or pipeline milking systems. In contrast, farms with a free-stall tend to be larger and mainly use milking parlours or automatic milking systems [10]. However, these are only some of the differences between the two housing systems. Given that large herds are usually housed in free-stall housing systems [11], the majority of the risk factor analyses for mastitis carried out recently have favoured this type of housing system. In order to avoid possible confounding biases due to associations between the type of housing system and a broad range of management-related risk factors, and to be able to specifically address the possible problems within each system, separate risk factor analysis for farms with a tie-stall and for farms with a free-stall (similar to a stratified study design and analysis) are considered necessary.

The main objectives of this study were to (1) estimate the annual incidence of clinical mastitis and the annual proportion of milk samples with a high SCC (above 200,000 cells/ml) in a random selection of Swiss dairy farms and (2) determine associations between on farm management practices and both mastitis outcomes separately for tie-stall and free-stall housing systems.

## Methods

### Target population and sample

Criteria for dairy farms to participate in the study were: (1) regular marketing of milk throughout 2010, (2) an average herd size >10 cows in 2010, and (3) a known email address. As of January, 2011, 65% of the Swiss dairy farms had a functional email address, with the proportion continuously increasing (personal communication from the Swiss national milk quality program, TSM Trust Ltd). The target population included 22’141 dairy farms with an average herd size of 24 dairy cows. The sample size was initially derived for a previous study in which the main objective was to provide a general overview of the main prophylactic measures used by Swiss dairy farmers in order to prevent diseases [10]. These sample size calculations indicated that at least 645 farms would be in order to estimate the prevalence on a response of interest (such as the proportion of farms implementing a certain preventive measure) of 50% from a finite target population of 22'141 dairy farms with an absolute error of 5% and 99% confidence. Considering an expected response rate of 30%, the minimum questionnaire sample size was set to 2,200 farms. Eventually, using a stratified (by Swiss canton) random sampling approach, 2,285 dairy farms (with stratum sample sizes proportional to the cantonal cattle population) was drawn from the available sampling frame using computer-generated random numbers [10]. All available responses from that survey were used for the current risk factor study.

### Data collection

For collecting data about general and prophylactic management practices as well as clinical mastitis occurrence, an online questionnaire was designed with a group of experts (including policy makers, veterinarians, agricultural advisors and dairy farmers). The questionnaire was implemented using the open source survey application LimeSurvey (http://www.limesurvey.org). It included 68 questions divided into 10 main sections (Table 1) and was offered in German, French and Italian. The questionnaire was pre-tested with 40 master students from the Swiss School of Agricultural, Forest and Food Sciences that had a dairy farming background. The final version was sent by email to the farmers in March 2011, with two follow-up reminder emails sent to all non-responders two and three weeks after the initial sending.

**Table 1:** Variables tested in the univariable analyses for associations with clinical mastitis and high milk somatic cell counts in a random sample of Swiss dairy farms with >10 cows

**Topic**	**Variables**
Demographic data	Farmers age, agricultural education, continuous agricultural training, language, farming as the main source of income
Farm characteristics	Agricultural zone, average milk production per cow in 2010, organic farming, affiliation to federal incentive programs
Farm management	Dairy cow replacement system, measures before purchasing a dairy cow, dairy cattle that spend the summer in alpine pastures, vaccination, parasite prophylactic measures, homeopathic treatments, herbal medicine treatments, claw trimming frequency
Stall characteristics	Type of stall, cow trainer†, type of flooring‡, flooring material‡, lying system‡, lying surface, bedding substrates, lying surface cleaning, feeding trough cleaning, drinking trough cleaning
Milking practices	Milking system, somatic cell count controls frequency, milk samples for bacteriological analysis taken from cows with clinical or subclinical mastitis
Drying off practices	Cessation of milking, milk samples for bacteriological analysis taken at drying off, California Mastitis Test (CMT) carried out at drying off, dry cow antibiotic treatments, dry cow internal teat sealant application, dry cow udder controls
Hygiene measures taken at milking	Hand washing before milking, clean clothing, apron, rubber gloves, hands cleaned during milking, no special measure, fore-milking, teat cleaning, teat cleaning material, fresh teat cleaning material used after each cow, post milking teat disinfection, cows are prevented from lying down after milking, strategy for milking CMT positive cows, teat cup liner material, teat cup liners changing frequency
Feeding practices	Written feeding plan, feed analysis carried out, type of feed ration for dairy cows, transit feed given before calving, body condition of dairy cows at calving in comparison with drying off, body condition score is monitored, number of concentrate intakes per day at the beginning of the lactation
Peripartum measures	Calving pen available, clean bedding material, calving environment is cleaned and disinfected, intervention at calving is normally avoided, hands and arms are cleaned if intervention is required, tail/vulva and surroundings are cleaned, mechanical obstetricians available, ropes or chains available, no special peripartum measure
Genetic selection	Most important characteristics when selecting a bull for artificial insemination

^†^Only applies to farms with a tie-stall.

^‡^Only applies to farms with a free-stall.

Responses were anonymous except when respondents provided their personal contact details at the end of the survey. With that they gave permission to retrieve milk production and reproduction data from the respective breeding organisations. Alternatively they were offered to manually enter the monthly farm level milk production and quality data for 2010 into the questionnaire. Farmers were informed that the data and information provided through the survey would be used in the context of a scientific study, and that summarized results would be presented in a report for the participating farmers as well as in lay-term and scientific publications. A written confidentiality agreement between the survey researchers and all official data providers (i.e., the national animal movement database operator, the milk quality records operator, and the cattle breeding organisations) ensured that information was exclusively used for this study, and that no individual farmer information was made public. A copy of the questionnaire (in German, French or Italian) is available from the corresponding author upon request.

In Switzerland, only human clinical studies and animal experiments have to be formally approved by regional (cantonal) ethical committees. In our case, questionnaire content, design and implementation procedure were approved by an individual oversight committee established by the Swiss Federal Veterinary Office that was composed of representatives of the veterinary services, cattle herd health services and the cattle industry.

### Population at risk

The actual number of cows per farm during 2010 was obtained from the Swiss Animal Movement Database on a monthly basis for the main site (location) of the farm. In order to derive the average number of cows per farm in 2010 (population at risk for the annual cumulative incidence), the original data for the months of June to September were excluded and assumed to be similar to the average of all other months. This was done to adjust for the fact that many farms let their cows graze on alpine pastures during this period, resulting in a substantial lower number of cows recorded in the animal movement database for the main location of the farm.

### Case definitions and frequency

Dairy farmers were asked to report the number of clinical mastitis cases they had observed during 2010. A specific case definition was provided in the questionnaire: a case of clinical mastitis was any cow with abnormal milk and/or visible or palpable changes in a quarter. Treatments of such animals have to be recorded in the mandatory farm treatment records. In these records, also the indication for the treatment is a mandatory component. The within-herd clinical mastitis incidence (ICM) was calculated as the number of clinical mastitis cases reported by the farmer in 2010 divided by the average number of cows per farm throughout 2010 and expressed as the number of farmer-reported cases of clinical mastitis per 100 cows and year. Given the nature of the denominator we considered this to be a cumulative incidence.

Somatic cell count (SCC) measurements from the routine test day recordings were provided by the three Swiss dairy cattle breeding associations (Swiss Brown Cattle Breeders’ Federation, Holstein Breeders’ Federation and Swissherdbook) for those farmers that had given their agreement in the questionnaire electronically. The average annual within-herd proportion of high SCC events (PSCC) was calculated as the sum of all test day records with an elevated (≥200,000 cells/ml) SCC per herd throughout 2010 divided by the sum of all test day records available in the year of study. The 200,000 cells/ml cut-off value was selected to minimize the diagnostic error [12].

### Classification of farms by housing system

In order to classify each farm by the predominant housing system, a question was included in the questionnaire that recorded whether the majority of cows on the respective farm were kept in a tie-stall or a free-stall. It was assumed that the farmer knew the difference between the two housing systems.

### Statistical analyses

Responses from the online questionnaire were downloaded from the LimeSurvey tool in MS Excel. The statistical software packages NCSS 2007 (NCSS LLC; Kaysville, Utah; http://www.ncss.com) and STATA 12 (StataCorp LP; College Station, Texas; http://www.stata.com) were used for the statistical analysis. Non-parametric Mann–Whitney U and Wilcoxon rank-sum tests were used to determine whether the ICM and the PSCC differed significantly between tie-stalls and free-stalls. The *X*^*2*^ tests were additionally used to determine differences in the overall proportion of farms that had reported clinical mastitis cases between tie-stall and free-stall housing systems. *P* values ≤ 0.05 were regarded as significant.

For the risk factor modelling, all continuous potential risk factors were recoded into two categories using the median of farms within the respective housing system as the cut-off value. Because of the frequently observed overdispersion of clinical mastitis data from dairy herds [13] - also present in this dataset when running explorative Poisson regression models (details not shown) - a negative binomial regression approach was used. Four separate negative binomial models were built within STATA to separately evaluate the effect of management practices on ICM and PSCC counts in the two housing systems. The natural log transformation of (a) the number of cows and (b) the total number of SCC measurements were entered as an offset into the respective models to account for different herd sizes. Variables with a *P-*value <0.1 in the univariable negative binomial regression models were considered as potential candidates for the multivariable selection process. The numeric correlation between all candidate variables selected for a model was assessed using a Spearman rank correlation matrix. To avoid multi-collinearity, the most biologically relevant variable was retained for the multivariable model if two variables had an absolute Spearman rank correlation coefficient >0.5 [14].

For the multivariable regression model, an automated stepwise forward selection and a backward elimination approach was performed. Final models were run on those records for which complete information on all variables in the model were available. The overall *P-*value for variable inclusion in the final model was set at P<0.05. Incidence rate ratios and 95% confidence intervals (CI) for risk factors associated with ICM or HSCC were estimated separately for tie-stall and free-stall housing systems. In the final multivariable models, individual IRRs were corrected for the confounding effect of the other factors in the model. Interactions between predictive variables were not further considered.

## Results

### Response rate

More than 50% of the contacted farmers returned a questionnaire but the item response varied from 35 to 53%, depending on the question [10]. Questions related to type of housing system and number of clinical mastitis cases treated in 2010 were completed by 43% (n=979) of the dairy farmers. Out of these farms, 587 had tie-stall barns and 392 had free-stall barns. Of those, 680 (412 with tie-stalls and 268 with free-stalls) belonged to a Swiss breeding association and had therefore composite SCC data available; they were included in the further analysis. Most of these farms (60%) belonged to Swissherdbook, 37% to the Swiss Brown Cattle Breeders’ Federation and 17% to the Holstein Breeders’ Federation.

### Dairy herd characteristics and mastitis occurrence

The median herd size was 24 cows (range: 11–71) for farms with a tie-stall and 37 cows (range: 11–128) for farms with a free-stall. The median number of cow level SCC tests per farm carried out in 2010 for tie-stalls was 164 (range: 14–741) and 265 (range: 40–941) for free-stalls.

**Figure 1:**
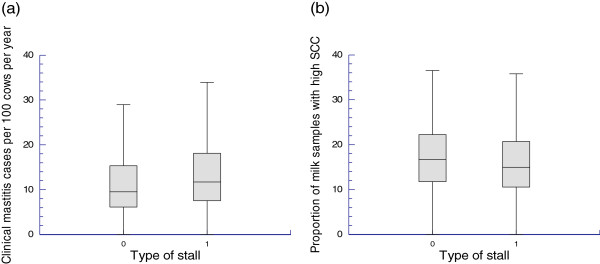
**Box and whisker plot of (a) farmer-reported cases of clinical mastitis per 100 cows per year (ICM) and (b) proportion of milk samples with a high somatic cell count (PSCC).** Data from a random sample of Swiss dairy farms with >10 cows in 2010. Type of stall: 0 = free-stall, 1 = tie-stall.

A median of 11.6 cases of clinical mastitis per 100 cows in 2010 was reported by the dairy farmers (95% confidence interval (CI); 10.9-12.3). This estimate was significantly higher for farms with a tie-stall (median 12.4) than for farms with a free-stall (median 9.9, *P*<0.001) (Figure 1). The proportion of farmers reporting one or more cases of clinical mastitis in their herds was 94.6%. This proportion was higher in free-stalls (96.6%) as opposed to tie-stalls (93.3%, *P*=0.02).

The median PSCC for 2010 was estimated to be 16.1% (95% CI; 15.3-16.8). Farms with a free-stall had a significantly higher proportion of PSCC samples (median 16.9%) than farms with a tie-stall (median 15.3%; *P*=0.04) (Figure 1).

### Risk factor analysis

Eight factors were significantly associated with the ICM on the 432 dairy farms with tie-stalls (Table 2). Within the 204 free-stall farms, ten factors were significantly associated with the IRM (Table 3).

**Table 2:** Final multivariable negative binomial regression model between management practices and clinical mastitis (incidence rate ratios (IRR) and 95% confidence intervals (CI)) from 432 Swiss dairy farms with a tie-stall and >10 cows in 2010

**Factor**	**Category**	**IRR (95% CI)**	***P-*value**
Farmers age	>45	0.81 (0.71-0.93)	0.003
	≤45	1.0	-
Production zone	Mountain	0.73 (0.62-0.85)	<0.001
	Hill	0.91 (0.75-1.09)	0.32
	Valley	1.0	-
Dairy herd replacement system	Buying cows	1.27 (1.08-1.50)	0.004
	Other†	1.0	-
Prophylactic measures taken when purchasing a dairy cow	Never purchase	0.83 (0.71-0.98)	0.02
	Other‡	1.0	-
Milking system	Bucket	1.21 (1.04-1.41)	0.01
	Pipeline	1.0	-
Teat cleaning material	Paper towel	1.19 (1.04-1.37)	0.01
	Other§	1.0	-
Feeding system	Cow specific	0.81 (0.66-0.99)	0.04
	Other^¶^	1.0	-
Intervention at each calving	No	0.87 (0.75-0.99)	0.04
	Yes	1.0	-

^†^Buying heifers, own rearing, contract rearing.

^‡^California Mastitis Test, milk samples taken for bacteriological culturing, somatic cell count data from breeding associations is checked, claw condition is checked.

^§^Wood wool, washable towel, wet towels with disinfectant, wet towels without disinfectant, no material.

^¶^The same for all cows, different for lactating and dry cows, different for different production groups.

**Table 3:** Final multivariable negative binomial regression model between management practices and clinical mastitis (incidence rate ratios (IRR) and 95% confidence intervals (CI)) from 204 Swiss dairy farms with a free-stall and >10 cows in 2010

**Factor**	**Category**	**IRR (95% CI)**	***P-*value**
Milk yield	High (> 6,122 kg)	1.25 (1.03-1.52)	0.01
	Low (≤ 6,122 kg)	1.0	-
Somatic cell count controls	At longer intervals	1.44 (1.13-1.84)	0.003
	≤2 times/month	1.0	-
Bacteriological test carried out for clinical mastitis	Never	0.61 (0.42-0.88)	0.01
	Always/occasionally	1.0	-
Dry cow udder controls during the dry period	Yes	0.67 (0.54-0.83)	<0.001
	No	1.0	-
Teat cleaning material	Washable towel	1.53 (1.04-2.25)	0.02
	Other†	1.0	-
Body condition scoring	No	0.74 (0.62-0.90)	0.002
	Yes	1.0	-
Important characteristic when selecting a bull for artificial insemination	Total merit values	1.57 (1.17-2.11)	0.002
	Other‡	1.0	-
Clean bedding material at calving	Yes	1.72 (1.24-2.38)	0.001
	No	1.0	-
Hands and arms are cleaned if intervention is required at calving	Yes	0.75 (0.61-0.93)	0.009
	No	1.0	-
Ropes or chains available at calving if required	Yes	1.37 (1.08-1.75)	0.009
	No	1.0	-

^†^Paper towel, wood wool, wet towels with disinfectant, wet towels without disinfectant, no material.

^‡^Milk yield, fat and protein content of milk, somatic cell count in milk, exterior, ease of calving, length of productive life, milking speed/persistence of lactation, fertility of the dam, beef production.

Six factors were significantly associated with the annual herd level HSCC incidence on the 263 dairy farms with a tie-stall (Table 4) while only two factors were significantly associated with the annual herd level HSCC incidence on the 219 dairy farms with free-stalls (Table 5).

**Table 4:** Final multivariable negative binomial regression model between management practices and high somatic cell counts (incidence rate ratios (IRR) and 95% confidence intervals (CI)) from 263 Swiss dairy farms with a tie-stall and >10 cows in 2010

**Factor**	**Category**	**IRR (95% CI)**	***P-*value**
Type of farming	Organic	0.72 (0.57-0.90)	0.005
	Conventional	1.0	-
Prophylactic measures taken when purchasing a dairy cow	Never purchase	0.87 (0.78-0.98)	0.02
	Other†	1.0	-
Type of stall	Open/cold	1.65 (1.19-2.28)	0.002
	Closed/warm	1.0	-
Lying surface	No mat	0.82 (0.65-1.03)	0.09
	Comfort mat	0.74 (0.62-0.89)	0.002
	Rubber mat	1.0	-
Water trough cleaning routine	At longer intervals	1.17 (1.03-1.33)	0.01
	Daily	1.0	-
Feed analysis carried out	Never	1.18 (1.06-1.32)	0.003
	Regularly/occasionally	1.0	-

^†^California Mastitis Test, milk samples taken for bacteriological culturing, somatic cell count data from breeding associations is checked, claw condition is checked.

**Table 5:** Final multivariable negative binomial regression model between management practices and high somatic cell counts (incidence rate ratios (IRR) and 95% confidence intervals (CI)) from 219 Swiss dairy farms with a free-stall and >10 cows in 2010

**Factor**	**Category**	**IRR (95% CI)**	***P-*value**
Hands and arms are cleaned if intervention during calving is required	Yes	0.81 (0.70-0.93)	0.003
	No	1.0	-
Important characteristic when selecting a bull for artificial insemination	Beef production	0.66 (0.47-0.93)	0.02
	Other†	1.0	-

^†^Milk yield, fat and protein content of milk, somatic cell count in milk, exterior, ease of calving, length of productive life, milking speed/persistence of lactation, fertility of the dam, total merit.

## Discussion

In this study, estimates of annual ICM and the proportion of HSCC in farms with tie-stall and free-stall housing systems in Switzerland were derived. In addition, farm-level management factors significantly associated with these outcomes in the two systems were identified. Although cautious interpretation is needed, the results give valuable insight in the current risk factors for mastitis in Switzerland. This information can be used to prioritize daily prophylactic dairy herd health practices at dairy farms by veterinarians and other udder health advisors and advices being given in national udder health programs.

### Clinical mastitis incidence

The farmer-reported ICM in this study (11.6 cases per 100 cows per year) was lower than in other recent studies with census data or a cross-sectional design carried out in Denmark [15], Norway [16], England and Wales [17], Canada [5] and The Netherlands [18]. It is important however to distinguish that in the current study farmers were asked to report the number of cows affected rather than the number of quarters affected.

Clinical mastitis frequency reported by the farmer most likely suffered from misclassification bias, which can work in two directions: (a) individual clinical mastitis cases might have been missed. Also some farmers might have recalled clinical mastitis events at the cow level rather than the case level. Both instances would have resulted in an underestimation; (b) farmers could have reported conditions other than CM. Also, when using mastitis cases instead of cows, there is a possibility that some cows are treated several times (even within the same mastitis case) and that is recalled by the farmer. These instances would have resulted in an overestimation of the ICM. Unfortunately it is not possible to check direction and magnitude of the reporting bias, but we consider it more likely that the direction was towards underreporting. If that assumption is correct the ICM estimates are too low, and the point estimates of all risk factor associations are biased towards the null hypothesis (type 2 error). Nevertheless, other studies [19] indicate that the influence of such reporting biases of cases is relatively small. In addition, studies carried out in the Nordic countries [20,21] point out that the ICM reported by farmers’ data is actually higher than the official disease recording system which is collected by the veterinarians, indicating such systems might be quite sensitive.

The extrapolation of number of cows present on farms that practiced alpine pasturing for the months of June to September was considered necessary to reduce bias in the denominator (population at risk) for the ICM estimates. Cows that are on alpine pasture are assigned a different location identifier in the animal movement database during that time. This results in substantially lower number of cows for the original farm location during the alpine grazing season and therefore in an underestimation of the population at risk for those farms. In consequence, clinical mastitis incidence would have been overestimated - given that clinical mastitis cases were still seen and recorded during milking on alpine pastures.

### High somatic cell counts

Free-stall housing systems have been reported to have lower SCC [7]. Nevertheless, the results of this study showed that in Switzerland, the average mean SCC was higher for farms with a free-stall than for farms with a tie-stall. This result is consistent with other studies carried out in Switzerland [22] and in Finland [23]. In addition, a study carried out in Norway indicates that herds with a tie-stall have a significantly higher clinical mastitis rate than herds with a free-stall [8] which is comparable to the results of this study.

### Risk factors

Within tie-stall farms, higher age of dairy farmers was associated with a decrease of IRCM. One possible interpretation is that older farmers have more experience, indicating that the knowledge acquired over the years could play an important role in udder health prevention. However, the observed effect could also be generated by older farmers being less stringent in diagnosing cases of clinical mastitis when compared to younger farmers, resulting in differential under-reporting.

Farms with a tie-stall on mountainous production zones showed to have a lower ICM when compared to valley located tie-stall farms. The reason for this association is difficult to explain. However, a risk factor analysis carried out previously in Switzerland for *Streptococci* species in quarter milk samples showed that *S. uberis* is significantly less prevalent on farms on mountainous production zones while, *S. dysgalactiae* is more prevalent [24]. These differences in bacterial species could explain why the ICM differs with the type of production zone. In addition, a further Swiss study indicated that milk somatic cell scores are higher in cows of valley situated farms [22] which is comparable to the results of this study.

The introduction of cows into a dairy herd is considered to be a risk factor for mastitis [25] which is consistent with the results of both risk factor analyses carried out for farms with a tie-stall in the current study. The similar variables “buying cows as a dairy herd replacement system” and “never purchasing dairy cows” were both contributing to the model for ICM in farms with a tie-stall housing system. They were only moderately correlated (r = 0.45). However, no clear difference was made between purchasing lactating cows or non-lactating heifers concerning the latter variable in the questionnaire. This could have resulted in farmers interpreting both variables differently. Nonetheless, the results of the final model indicated that both variables related to dairy cow replacement are independently linked to udder health. They also pointed in the same direction.

Studies showed that there is a tendency for better udder health on organic farms compared to conventional farms [26-28], although a study carried out in Switzerland in 2003 [29] did not identify any significant differences. In the current study, organic farms with a tie-stall showed to have a lower proportion of high SCC cows compared to conventional farms with a tie-stall. In addition, having a closed or warm stall was associated with lower SCC levels on farms with a tie-stall, although this should nevertheless be interpreted with caution as the majority (97.4%) of farms with a tie-stall in Switzerland tend to have a closed stall as opposed to an open stall [10]. With respect to the lying surface, the use of comfort mats was associated with lower SCC levels on farms with a tie-stall. The advantages of this type of surface with respect to udder health are also indicated in other studies [30-32].

Regarding milking practices, bucket milking as opposed to pipeline milking showed to be associated with a higher ICM on farms with a tie-stall. It could be argued that farms with bucket milking have older milking facilities and stalls when compared to farms with pipeline milking resulting in a higher risk for infection. In addition, with respect to the hygiene measures taken during milking, the type of material used for teat cleaning was associated with the ICM. The use of paper towels on farms with a tie-stall and washable towels on farms with a free-stall as opposed to other teat cleaning materials such as wood wool, wet towels with disinfectant, wet towels without disinfectant or no material showed to be associated with ICM. A study carried out in Spain [33] associates the use of paper towels (as opposed to cloth towels) to a lower milk SCC. However, to our knowledge, no studies have been conducted that were able to identify the causal relationship between the type of cleaning material and the ICM at dairy herds. It therefore remains speculation which type of cleaning material is preferable concerning udder health performance.

The use of a calving pen as a hygienic measure taken at calving [34] as well as cleaning it after each calving [7] are factors which are associated with a lower PSCC. It has also been described how different bedding materials can play an important role in the transmission of pathogens to the udder [35,36]. The results from this study have also shown how measures taken at calving are associated with mastitis. First, the use of clean bedding material at calving was associated with a higher ICM in farms with a free-stall although this is a well described prophylactic measure to be taken at calving. For this reason it could be interpreted that farmers which have more problems with mastitis postpartum tend to compensate by providing clean bedding material at calving. Second, an association between intervening at calving and a higher ICM was identified for both tie-stall and free-stall housing systems. This could be an indicator for the ease of calving. Cows giving birth without any farmers’ assistance may have a better udder health than cows that do need assistance because they are more vital, thereby also reducing the time in which they have contact with contaminated bedding material in a period where they are highly susceptible for new intramammary infections [37]. Finally, the observation that washing hands at calving was associated with lower ICM levels and a lower PSCC in free-stall might be indicators for an overall improved hygiene in the entire farm. However, a causal relationship cannot be excluded. Further studies are needed to confirm these results.

Milk yield is positively correlated with a higher risk of clinical mastitis [25,38-40]. This is consistent with the higher ICM that was observed in the current study for high producing farms with a free-stall.

A cow specific feeding system (versus a standard ration for all cows, different rations for lactating and dry cows, or different rations for different production groups) as well as carrying out feed analysis showed to be associated with a lower ICM and with a lower SCC respectively for farms with a tie-stall. In addition, daily water trough cleaning in farms with a tie-stall was also associated with a lower milk SCC. This association was also found in a survey carried out in the USA [41]. On the other hand, the practice of body condition scoring was associated with a higher ICM for farms with a free-stall, even though the dairy health problems caused by an inadequate body condition are well described in the literature [39]. One possible explanation for this association could be that farmers with a higher ICM in their herd score the body condition of their cows during the lactation and the dry period more frequently in order to control mastitis and other diseases.

This study found associations between an increased ICM and the use of milk bacteriological tests on dairy farms with a free-stall. The reason for this association could be that farms with a higher ICM need to carry out more milk bacteriological tests to identify the causative pathogens. In addition, dry cow udder controls were associated with a lower ICM for farms with a free-stall, which is in agreement with the lower bulk milk SCC observations in farms which daily check the udder for mastitis during the dry period [42].

With respect to the genetic selection and the most important characteristics when selecting bulls for artificial insemination, total merit values showed to be associated with a higher ICM in farms with a free-stall housing system. Moreover, selecting for beef production was associated with a lower milk SCC for farms with a free-stall. Although the heritability estimates of CM and high SCC are generally low [43], selection experiments have shown that CM cases can be reduced when farmers breed for mastitis resistance while CM frequency increased when they breed for increased protein yield [44]. An improved genetic basis for CM resistance is also assumed to be one of the key factors for the udder health improvement resulting from the Norwegian mastitis control program [45].

The possible influence of the different contagious or environmental udder pathogens causing intramammary infection was not taken into account in the current risk factor analyses. Therefore, future studies should account for the main type of pathogens involved in the cases of clinical mastitis or high SCC as the association with the different management practices could vary considerably for each pathogen [46].

Given the cross-sectional design of the study, the associations identified should not automatically be considered as causal. Therefore, the results should be used with caution when transferred into dairy disease prevention programs.

## Conclusions

The results from this study revealed that different dairy farm characteristics, milking practices as well as feeding management, measures taken at calving, and genetic characteristics showed some level of association with udder health. The statistical associations that were found between the management practices surveyed and both mastitis manifestations varied considerably between the two different types of housing systems. This is considered to be the consequence of the complex multi-causal system responsible for mastitis at the herd level. In consequence, future udder health campaigns should not only consider major differences between housing systems when issuing prevention recommendations but ideally have to consider each farm as an individual biological system that requires specific measures to control a mastitis problem.

## Abbreviations

ICM: Cumulative incidence of clinical mastitis on a farm; SCC: Composite somatic cell count; PSCC: Proportion of high (> 200’000 cells/ml) SCC measurements in 2010 on a farm; CI: Limits of the respective confidence interval.

## Competing interests

The authors declare that they have no competing interests.

## Authors’ contributions

PG made substantial contributions to design and implementation of the study, collected and analyzed the data, and drafted the manuscript as part of her dissertation project. BB & MR made substantial contributions to the concept and design of the study, assisted in the data analysis and interpretation of the results and were involved in critically revising the manuscript. SK and MD developed the concept of the project and supervised the implementation, data collection and statistical analysis. They were involved in critically revising the manuscript. MD was mainly responsible for the revision of the manuscript. All authors have given their final approval of the version to be published.
